# Characterising hospitalisation risk for chronic obstructive pulmonary disease exacerbations: Bedside and outpatient clinic assessments of easily measured variables

**DOI:** 10.1177/14799731231211852

**Published:** 2023-11-07

**Authors:** Joshua Heerema, Sarah Hug, Natasha Bear, Kylie Hill

**Affiliations:** 1 Physiotherapy Department, 5728 Sir Charles Gairdner Hospital, Nedlands, Australia; 2 Curtin School of Allied Health, 1649 Curtin University, Perth, Australia; 3 Physiotherapy Department, Royal Perth Hospital, Perth, Australia; 4 Institute of Health Sciences, Notre Dame University, Perth, Australia

**Keywords:** Chronic obstructive pulmonary disease, hospitalisation, exacerbation, risk

## Abstract

**Objective:**

To identify the characteristics of people with chronic obstructive pulmonary disease (COPD) who require hospitalisation for exacerbations.

**Methods:**

People with COPD were recruited either during hospitalisation or from out-patient respiratory medicine clinics. Hospital admissions were tracked throughout the 5-months recruitment period. For participants who were admitted, hospital readmissions were tracked for at least 30 days following discharge. Participants were grouped as either needing; (i) no hospital admission during the study period (no admission; ø-A), (ii) one or more hospital admissions during the study period but no readmission within 30 days of discharge (no rapid readmission; ø-RR) or (iii) one or more hospital admissions with a readmission within 30 days of discharge (rapid readmission; RR).

**Results:**

Compared with the ø-A group (*n*=211), factors that independently increased the risk of ø-RR (*n*=146) and/or RR (*n*=57) group membership were being aged >60 years, identifying as an Indigenous person (relative risk ratio, 95% confidence interval 7.8 [1.8 to 34.0]) and the use of a support person or community service for activities of daily living (1.5 [1.0 to 2.4]. A body mass index ≥25 kg/m^2^ was protective.

**Conclusions:**

Variables recorded at the bedside or in clinic provided information on hospitalisation risk.

## Introduction

Chronic obstructive pulmonary disease (COPD) is a common condition, and is the most prevalent chronic respiratory condition worldwide.^1^ Across Australia, approximately one in 13 Australians aged ≥40 years have airflow obstruction that is moderate or greater in severity.^2^ In 2022, COPD accounted for 3.7% of the total disease burden in Australia.^3^ The cost to the Australian health system associated with COPD was an estimated AUD$994.8 million in 2019-20, representing 21% of disease expenditure on respiratory conditions.^3^ Chronic obstructive pulmonary disease is characterised by periods of clinical stability that are interrupted by periods of acute worsening, known as exacerbations. A large dataset (*n* = 3769) from a 3 year, multicentre, randomised parallel group study conducted across 42 countries revealed that over a 12-month period, more than half the sample of participants with moderate to very severe COPD had experienced an exacerbation.^4^ The costs associated with exacerbations that require hospitalisation are substantial,^4^ with the greatest cost driven by people with severe disease who experience frequent exacerbations.^5^ Indeed, in people with COPD, a hospitalisation in the previous 12 months is commonly reported risk factor for another hospitalisation,^6^ and the clinical course after two severe exacerbations is characterised by a rapid decline in health status and high mortality.^7^

Identifying the characteristics of those at higher risk of hospitalisation may allow healthcare providers to prioritise access to interventions that have been shown to minimise readmission risk and reduce healthcare costs. To date, studies that have explored the characteristics associated with increased hospitalisation risk have used measures that may not be commonly available to clinicians at the bedside, such as spirometry^6^ and questionnaire-based measures of feelings of depression and anxiety and health-related quality of life (HRQoL).^8–10^ For example, an audit of clinical practice for people hospitalised with an exacerbation of COPD found that measures of spirometry were performed on less than one in 4 cases.^11^ The current study addresses this issue by exploring whether or not variables that are readily available to clinicians (at the bedside) can predict hospitalisation risk. This may guide clinicians to prioritise those who appear to be at greatest risk of hospitalisation to access to interventions that have been shown to reduce admissions, such as pulmonary rehabilitation programs (PRPs).^12^

For this study, we sought to answer the following research question: in people with COPD who are known to a tertiary hospital in Perth, Western Australia, what, if any, measures that are generally noted during clinical practice differ between people grouped according to their hospitalisation needs during a 5-months follow-up period?

## Methods

This study presents a pragmatic and opportunistic analyses of an existing dataset collected as part of a larger prospective observational study, which has been published elsewhere^13^ and had received approval from all relevant Human Research Ethics Committees (project number RGS0000003704). All participants gave written informed consent prior to participation. For the larger study, people with COPD were sequentially approached to participate if they were fluent in English and were either hospitalised for a suspected exacerbation of COPD or were attending an out-patient respiratory medicine clinic at one of the three tertiary hospitals in Perth, Western Australia. As the larger study sought to report practices related to the implementation of pulmonary rehabilitation programs (PRPs), participants were excluded if they were unlikely to be appropriate for one of these programs. Specifically, people were excluded if they had recently attended a PRP, were unable to ambulate independently, had evidence of cognitive impairment or inability to understand English, lived in supported residential care, or were deemed to be within the final 6 months of life. 

### Data collection

Recruitment occurred between August 2020 and February 2021. The 5-months follow-up period for this study was based on timelines dictated by the funding body for the larger study. Variables used in the current analyses were obtained by research staff from participants' medical records and participant interview (in person or via phone call). Data were collected using Research Electronic Data (REDCap) collection tools which are available on our Open Science Framework project page.^14^ Demographic variables comprised of participant age, body mass index (BMI), ethnicity, sex and smoking status. Further variables included lung function, pulmonary comorbid conditions (e.g. asthma), other comorbid conditions (e.g. cardiovascular disease [CVD]), social situation (e.g. whether they lived alone or with someone), whether or not participants received assistance at least once weekly with activities of daily living (ADLs), socioeconomic status (i.e. Index of Relative Socio-economic Advantage and Disadvantage [IRSAD]),^15^ and whether participants were referred to or attended a PRP. For all participants, hospital admissions were tracked from the point of recruitment to the conclusion of the recruitment period or for 30 days, whichever was longer. Any participant who required an admission was tracked for readmissions for a minimum of 30 days following discharge, using the hospital electronic systems.

### Quality control measures

All research staff completed standardised training prior to undertaking data collection. The REDCap database was screened twice weekly by the investigators to identify errors or omissions and the research staff were requested to amend these errors as soon as possible. Within the REDCap system, limits were set to minimise the entry of spurious data (e.g. height, was recorded in meters with a limit set at 2, which meant that research staff could not inadvertently enter this variable in centimetres). Conversion from imperial to metric measures was automated within REDCap. During analyses, data were plotted and examined for plausible values and spurious values were amended.

### Data management of statistical analyses

Data are reported as frequencies and proportions (categorical data) or means and standard deviations (continuous data). We adapted definitions used in a previous study^16^ and grouped participants into one of the following categories: (i) no hospital admission during the study period (no admission [ø-A]), (ii) one or more hospital admissions during the study period but no readmission within 30 days of discharge (no rapid readmission [ø-RR]) or (iii) one or more hospital admissions during the study period, with a readmission within 30 days of discharge (rapid readmission [RR]). Variables were compared between groups using multinomial logistic regression with group membership as the dependent variable. Multinomial logistic regression was chosen as it enables a model to have three outcomes categories, whereas simple logistic regression would only allow two categories. Ordinal logistic regression was not suitable as we could not meet the assumption of proportional odds. Unadjusted models were calculated for each variable, with the output expressed as relative risk ratios with their corresponding 95% confidence intervals (CI). Thereafter, a multivariable model was created through purposeful selection of variables using the technique of backwards elimination. The initial model included any variable with a likelihood-ratio test (LRT) less than 0.3. Variables that did not contribute to the model were eliminated one at a time. Contribution to the model was assessed through examination of the LRT for each variable and reviewing the impact of the removal of the variable on the other estimated coefficients. The final multivariable model presented was adjusted for duration of follow-up given the duration varied between participants.

All predictors had complete data, with the exception of forced expiratory volume in one second (FEV_1,_ % predicted), which was displayed in univariable analysis. A multivariable analysis was conducted using the reduced dataset with FEV_1_ expressed as percent predicted (% predicted), however FEV_1_ did not reach statistical significance. Missing data methods such as multiple imputation was not suitable as we could not confirm the data was missing at random. Data were analysed using Stata 16.1 (StataCorp, College Station, TX, USA), with statistical significance set as < 0.05.

## Results

**Figure 1.**
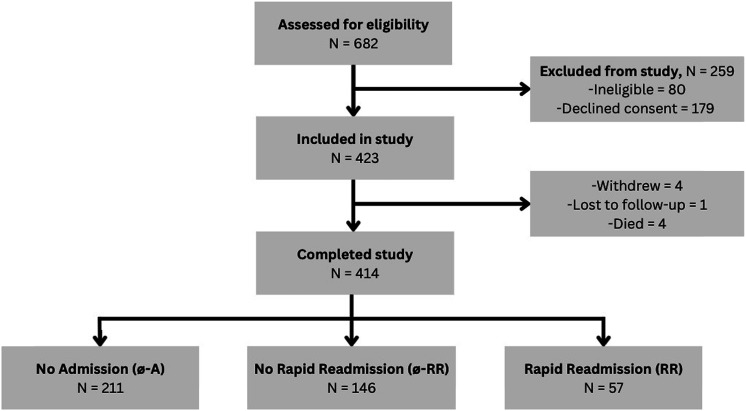
Flow of participants into the study.

The flow of participants into the study is summarised in Figure 1. Of the 682 people screened to participate in the larger study, 259 did not meet study criteria or declined to participate. Of the remaining 423 participants, data collection for this study was complete for 414 participants. Data were missing for measures of lung function: FEV_1_ litres (*n*=52), FEV_1_ % predicted (*n*=51), forced vital capacity (FVC) in litres (*n*=53), FVC % predicted (*n*=52) and diffusing capacity of lungs for carbon monoxide (DL_co_) % predicted (*n*=112).

### Participant characteristics

A total of 211 (51%), 146 (35%) and 57 (14%) participants were classified as ø-A, ø-RR and RR, respectively. Within our sample, of those who were admitted at least once during the recruitment period (*n* = 203), 57 (28%) required another admission within 30 days of discharge. Participant characteristics are presented in Table 1.

**Table 1.** Participant characteristics.

	No admission (ø-A)	No rapid readmission (ø-RR)	Rapid readmission (RR)
Demographic variables				
Age (yr)	67.0 (10.6)	71.7 (9.0)	71.8 (9.5)
BMI (kg/m^2^)	27.7 (6.9)	28.2 (8.0)	26.5 (9.0)
Ethnicity				
Indigenous	3 (1.4%)	8 (5.5%)	7 (12.3%)
Caucasian	202 (95.7%)	134 (91.8%)	50 (87.7%)
Other	6 (2.8%)	4 (2.7%)	0 (0.0%)
Sex				
Male	119 (56.4%)	81 (55.5%)	27 (47.4%)
Smoking status				
Current smoker	68 (32.2%)	47 (32.2%)	24 (42.1%)
Ex-smoker	138 (65.4%)	97 (66.4%)	32 (56.1%)
Never	5 (2.4%)	2 (1.4%)	1 (1.8%)
Social factors				
IRSAD				
1	50 (23.7%)	25 (17.1%)	11 (19.3%)
2	39 (18.5%)	30 (20.5%)	9 (15.8%)
3	45 (21.3%)	32 (21.9%)	12 (21.1%)
4	35 (16.6%)	16 (11.0%)	6 (10.5%)
5	42 (19.9%)	43 (29.5%)	19 (33.3%)
Receives assistance with ADLs				
No	137 (64.9%)	75 (51.4%)	20 (35.1%)
Yes	74 (35.1%)	71 (48.6%)	37 (64.9%)
Social situation				
Lives alone	66 (31.3%)	58 (39.7%)	20 (35.1%)
Lives with ≥ one other person	145 (68.7%)	88 (60.3%)	37 (64.9%)
Pulmonary comorbid conditions				
Additional lung disease	72 (34.1%)	40 (27.4%)	18 (31.6%)
Asthma	67 (31.8%)	35 (24.0%)	11 (19.3%)
Bronchiectasis	15 (7.1%)	8 (5.5%)	6 (10.5%)
Lung cancer	15 (7.1%)	8 (5.5%)	4 (7.0%)
Other comorbid conditions				
Cardiovascular disease	128 (60.7%)	108 (74.0%)	43 (75.4%)
Diabetes	20 (9.5%)	22 (15.1%)	9 (15.8%)
GORD	66 (31.3%)	57 (39.0%)	20 (35.1%)
Indicator(s) of poor bone health	89 (42.2%)	67 (45.9%)	30 (52.6%)
Mental health disorder	78 (37.0%)	51 (34.9%)	19 (33.3%)
Osteoarthritis	47 (22.3%)	23 (15.8%)	6 (10.5%)
Lung function				
FEV_1_ (L)	1.4 (0.6)	1.2 (0.5)	1.1 (0.4)
FEV_1_ (% predicted)	53 (21)	46 (17)	44 (18)
FVC (L)	2.9 (0.9)	2.7 (0.9)	2.4 (0.7)
FVC (% predicted)	83 (20)	78 (20)	74 (20)
D_L_CO (% predicted)	59 (18)	52 (18)	51 (17)
Pulmonary rehabilitation				
Referred to PRP	56 (26.5%)	53 (36.3%)	15 (26.3%)
Attended a PRP	28 (13.3%)	32 (21.9%)	9 (15.8%)

Data are *n* (%) or mean (SD).

‘Additional lung disease’ captured any other diagnosis of a respiratory condition, other than COPD, asthma, bronchiectasis or lung cancer (e.g. interstitial lung disease, asbestosis).

‘Cardiovascular disease’ was defined as having evidence of any of the following: angina, arrhythmia, cardiomyopathy, congestive cardiac failure, ischaemic heart disease, myocardial infarction or stroke.

‘Ethnicity’ was grouped as Indigenous, Caucasian or other (e.g. Asian).

‘Indicators of poor bone health’ were defined as having any of the following; (i) self-reported problems with bone health, (ii) previous imaging for bone health, (iii) history of fragility fracture or, (iv) advised to use medication/supplements to optimise bone health.

The ‘Index of Relative Socio-economic Advantage and Disadvantage (IRSAD)’ index summarises information about the economic and social conditions of people and households within an area, including both relative advantage and disadvantage measures. The index is derived from attributes including burden of dependent persons, disabilities affecting independence, education, employment status and income. A low score (1) indicates relatively greater disadvantage and a lack of advantage in general, while a high score (5) indicates a relative lack of disadvantage and greater advantage in general.

**Definitions of abbreviations:** ADLs: activities of daily living; BMI: body mass index; DLCO: diffusing capacity for carbon monoxide; FEV_1_: forced expiratory volume in 1 s; FVC: forced vital capacity; GORD: gastro-oesophageal reflux disease; IRSAD: Index of Relative Socio-economic Advantage and Disadvantage; PRP: pulmonary rehabilitation program.

The variables that were significant in the unadjusted models are presented in Table 2. Compared with the ø-A group, the variables that increased risk the ø-RR group membership were advancing age, identifying as an Indigenous person, having evidence of CVD and receiving assistance with their ADLs. The risk of being in the ø-RR group decreased with increasing FEV_1_ (% predicted). Characteristics that separated the ø-A from the ø-RR were similar to those which separated the ø-A and RR groups. However, compared with the ø-A group, the risk of RR group membership was reduced in those who were overweight (BMI 25 to <30 kg/m^2^). Compared with the ø-RR group, the only variable that changed (reduced) the risk of RR group membership was being overweight or obese.

**Table 2.** Unadjusted multinomial logistic regression model.

Relative risk ratio (95% CI)				
Variables	Comparison 1	Comparison 2	Comparison 3	*p*-value for likelihood ratio
	ø-A vs ø-RR	ø-A vs RR	ø-RR vs RR	
Age (yr)				
40 to <60	1.0 (reference)	1.0 (reference)	1.0 (reference)	*p* < 0.001
60 to <70	3.0 (1.4 to 6.3)	1.6 (0.6 to 4.4)	0.5 (0.2 to 1.7)	
70 to <80	4.7 (2.2 to 9.9)	3.9 (1.5 to 10.2)	0.8 (0.3 to 2.5)	
80+	4.1 (1.8 to 9.4)	2.9 (0.9 to 8.7)	0.7 (0.2 to 2.4)	
Indigenous (yes)	4.0 (1.0 to 15.4)	9.7 (2.4 to 38.9)	2.4 (0.8 to 7.0)	*p* = 0.002
BMI (categories)				
Underweight (<18.5 kg/m^2^)	1.7 (0.7 to 4.0)	1.3 (0.4 to 3.7)	0.8 (0.3 to 2.2)	*p* = 0.001
Healthy weight (18.5 to <25 kg/m^2^)	1.0 (reference)	1.0 (reference)	1.0 (reference)	
Overweight (25 to <30 kg/m^2^)	0.9 (0.5 to 1.6)	0.2 (0.1 to 0.6)	0.2 (0.1 to 0.7)	
Obese (30+ kg/m^2^)	1.4 (0.9 to 2.4)	0.6 (0.3 to 1.2)	0.4 (0.2 to 0.9)	
Cardiovascular disease	1.8 (1.2 to 2.9)	2.0 (1.0 to 3.9)	1.1 (0.5 to 2.2)	*p* = 0.011
FEV_1_ (%pred)	0.98 (0.97 to 0.99)	0.98 (0.96 to 0.99)	0.99 (0.97 to 1.01)	*p* = 0.001
Assistance with ADLs (yes)	1.8 (1.1 to 2.7)	3.4 (1.9 to 6.3)	2.0 (1.0 to 3.7)	*p* < 0.001

Data are relative risk ratios and (95% confidence interval [CI]). For comparison 1 and 2, ø-A was the reference group. For comparison 3, ø-RR was the reference group.

The *p*-value represents the overall significance of the model for the predictor. It is an omnibus test that indicates that for at least one of the predictors, the regression coefficients are significant.

Additional variables trialled in the model (but not presented as they were not significant) were social situation (see Table 1), history of diabetes, history of osteoarthritis, whether they were referred to a PRP and whether they attended a PRP.

Sample size available for all variables was *n* = 414, except FEV_1_ (%predicted) for which *n* = 363.

**Definition of abbreviations:** ADLs: activities of daily living; BMI: body mass index; FEV_1_: forced expiratory volume in 1 s; ø-A: no admission; ø-RR: one or more hospital admissions but no readmission within 30 days of discharge; RR: one or more hospital admissions with a readmission within 30 days of discharge.

The variables retained in the adjusted models are presented in Table 3. The adjusted model produced similar findings to the unadjusted model, however a history of CVD and FEV_1_ (% predicted) were not retained as characteristics that separated the ø-A group from the ø-RR group.

**Table 3.** Adjusted multinomial logistic regression.

Relative risk ratios (95% CI)				
Variables	Comparison 1	Comparison 2	Comparison 3	*p*-value for likelihood ratio
	ø-A vs ø-RR	ø-A vs RR	ø-RR vs RR	
Age (yr)				
40 to <60	1.0 (reference)	1.0 (reference)	1.0 (reference)	*p* < 0.001
60 to <70	3.9 (1.7 to 8.7)	2.5 (0.8 to 8.0)	0.6 (0.2 to 2.2)	
70 to <80	6.1 (2.7 to 13.7)	6.0 (1.9 to 18.8)	1.0 (0.3 to 3.3)	
80+	5.3 (2.1 to 13.3)	3.6 (1.0 to 13.5)	0.7 (0.2 to 2.7)	
Indigenous (yes)	7.8 (1.8 to 34.0)	21.4 (4.4 to 103.1)	2.7 (0.8 to 8.9)	*p* < 0.001
BMI (categories)				
Underweight (<18.5 kg/m^2^)	1.6 (0.6 to 3.9)	1.2 (0.4 to 3.7)	0.7 (0.2 to 2.3)	*p* = 0.006
Healthy weight (18.5 to <25 kg/m^2^)	1.0 (reference)	1.0 (reference)	1.0 (reference)	
Overweight (25 to <30 kg/m^2^)	0.8 (0.4 to 1.4)	0.2 (0.1 to 0.5)	0.2 (0.1 to 0.7)	
Obese (30+ kg/m^2^)	1.3 (0.8 to 2.3)	0.5 (0.2 to 1.1)	0.4 (0.2 to 0.8)	
Assistance with ADLs (yes)	1.5 (1.0 to 2.4)	3.4 (1.7 to 6.8)	2.2 (1.1 to 4.4)	*p* = 0.002

Data are relative risk ratio and (95% confidence interval [CI]). For comparison 1 and 2, ø-A was the reference group. For comparison 3, ø-RR was the reference group. The displayed model was adjusted for covariates, plus duration of follow-up.

The *p*-value represents the overall significance of the model for the predictor. It is an omnibus test that indicates that for at least one of the predictors, the regression coefficients are significant.

Sample size available for all variables was *n* = 414.

**Definition of abbreviations:** ADLs: activities of daily living; BMI: body mass index; ø-A: no admission; ø-RR: one or more hospital admissions but no readmission within 30 days of discharge; RR: one or more hospital admissions with a readmission within 30 days of discharge.

## Discussion

This study presents an opportunistic analyses of a dataset of just over 400 people with COPD who were known to a tertiary hospital in Perth, Western Australia. The important findings of this study are: (i) of those who were admitted to hospital at least once during the recruitment period, approximately one in four required a readmission within 30 days of hospital discharge, (ii) compared with not having an admission, variables that increased the risk of being in the ø-RR or RR groups were advancing age, identifying as an Indigenous person and receiving assistance with their ADLs and, (iii) having a BMI above the healthy range reduced the risk of being in the ø-RR and the RR groups.

Understanding the “frequent exacerbator” phenotype has been an area for interest for more than a decade. The unadjusted logistic regression model suggested that compared with those who were not hospitalised, those who were hospitalised were characterised by worse airflow obstruction and a higher prevalence of CVD. Although these variables were not retained in the adjusted model, this finding is consistent with earlier work demonstrating that those who require hospitalisations for COPD are often characterised by greater clinical complexity.^6,17^ Our data reveals that following discharge for an exacerbation, approximately one in four will be readmitted within 30 days. Data collected during the ECLIPSE study suggests the readmission rate increases as the follow-up period is extended, such that at 90 days following discharge, approximately one in three people have been readmitted and within 180 days following discharge, one in two have been readmitted.^10^ The phenomenon of exacerbations clustering together has been described previously.^16^ Our study builds on earlier work by demonstrating that factors which can easily be ascertained by clinicians at the bedside, such as advancing age, identifying as an Indigenous person, receiving assistance with their ADLs and having a BMI <25 kg/m^2^ characterised those at increased risk of hospitalisation.

Our finding that advanced age and receiving assistance with ADLs were independent risk factors for hospitalisation corroborates earlier work that demonstrated standardised measures of frailty, such as the Clinical Frailty Scale, were associated with hospitalisation in people with COPD.^18^ Frailty has been identified as a characteristic associated with worse clinical outcomes such as in-hospital mortality and difficulty returning home in people with COPD,^19^ and may identify those who engage in the lowest levels of walking-based activity during an admission.^20^ A large dataset (*n* ∼ 6000) of people with a substantial smoking history or a diagnosis of COPD revealed that over a 3 to 5 years follow-up period, severe exacerbations resulted in a loss of muscle mass that was at least 2% greater than could be accounted for by variables such as age and sex.^21^ Of note, this loss was mitigated in those who participated in a PRP during the follow up period.

Although we recruited a small number of people who identified as an Indigenous person (*n* = 18) this was an important predictor of hospitalisation. Compared with non-Indigenous people, those who identified as Indigenous have a high prevalence of multiple chronic respiratory conditions, with almost half of those with evidence of airway disease (such as emphysema) having concurrent bronchiectasis.^22^ For Indigenous people with COPD there is limited access to culturally safe healthcare services, including PRPs,^23,24^ which likely contributes to a number of avoidable admissions each year.^25^

Our data also demonstrated that a BMI ≥25 kg/m^2^ was protective against hospitalisation. This is an interesting finding as few previous studies have explored the relationship between BMI and hospitalisation in people with COPD.^6^ Specifically, BMI has been associated with hospitalisation within a 12-month period,^26^ and a low BMI (≤21 kg/m^2^) is predictive of worse survival.^27^ The protective role that a BMI ≥25 kg/m^2^ has against hospitalisation may be explained, at least in part by preservation of muscle mass^21^ but also by the “obesity-paradox”. That is, in people with COPD who are characterised by similar degree of airflow obstruction, those who are overweight or obese have reduced operating lung volumes (i.e. less resting and dynamic lung hyperinflation) and experience less dyspnoea as equivalent levels of minute ventilation during cycle-based exercise.^28–30^ This change in respiratory mechanics may also ameliorate the onset of intolerable dyspnoea during exacerbation; the most distressing symptom that precipitates a hospital admission.^31^

These data suggest that older adults with COPD, who receive support to complete ADLs, especially those with a BMI <25 kg/m^2^, should be prioritised for interventions known to reduce hospitalisation risk. These include initiation of smoking cessation strategies, enrolment in PRPs, optimising pharmacotherapy and inhaler technique,^32^ and participation in community programs that aim to minimise readmission.^33–35^ Further, as a delay of >24 hours in the initiation of treatment for an exacerbation has been shown to double the odds of hospital admission,^36^ it is important to provide these people with the skills and confidence to implement a comprehensive action plan. Regarding PRPs, in older adults with COPD, who receive support to complete ADLs, and those with a BMI <25 kg/m^2^, it is likely that, in addition to aerobic exercise training, attention should be given to strategies that increase muscle mass such as resistance exercise and appropriate dietary support.

### Strengths and limitations

The main strengths of this study were that all individuals who met the criteria were sequentially approached to participate and that data collection was completed prospectively and corroborated using medical health records and participant interview. The weakness of this study was that the time period for follow-up was not standardised for all study participants. That is, someone recruited at the beginning of the study from an out-patient respiratory clinic had their hospitalisations tracked for 5 months, whereas someone recruited from these clinics in the final month of the study had their hospitalisations tracked for 30 days. This difference in follow-up duration means that we may have overestimated the proportion of people in the ø-A group. That is, if we had followed those who were recruited later in the study for longer, they may have exacerbated and thereby met the criteria to be in the ø-RR or the RR group. This means that the risk of being in the ø-RR or RR (compared with the ø-A group) attributed to advancing age, identifying as an Indigenous person and receiving assistance with their ADLs, although sensible, may be overestimated in our analyses and causality cannot be definitively established without robust evidence from large cohort studies. Of note however is that the estimates of the risk ratios were unchanged when the model was adjusted for differences in the time period available for follow-up.

Notwithstanding this consideration, our analyses showing that approximately one in four people with COPD were readmitted within 30 days of hospital discharge and that a BMI ≥25 kg/m^2^ was protective against readmission were unaffected by this potential limitation. We also acknowledge that the sample recruited for this study were those who appeared broadly appropriate for referral to a PRP. i.e., we excluded those who were from residential care and those who had previously completed a PRP and this may have introduced bias in our results. The sample size for the ø-RR and RR groups was somewhat modest, and we may have lacked power to detect small differences between these groups. These data were collected in people known to a tertiary hospital in Perth, Western Australia and this may limit the generalisability of our findings to populations outside Australia and those without access to tertiary hospital care (i.e. rural and remote communities).

## Conclusion

The analyses presented in the current study should be considered exploratory. They suggest that in people with COPD who were known to a tertiary hospital in Australia, and appeared appropriate for referral to PRP, variables easily recorded during a patient interview can provide information on hospitalisation risk. However, this contention requires further research. The clinical implication of this finding is that older adults with COPD, who receive support to complete ADLs, especially those with a BMI <25 kg/m^2^, should be prioritised for enrolment in interventions known to reduce hospitalisation risk such as pulmonary rehabilitation programs which includes education regarding the use of comprehensive action plans. There is an urgent need to develop culturally appropriate PRPs for Indigenous people.
